# Knee Loading With Blood Flow Restriction Can Enhance Recovery After Total Knee Arthroplasty

**DOI:** 10.7759/cureus.37895

**Published:** 2023-04-20

**Authors:** Clément De Renty, Florian Forelli, Jean Mazeas, Georgios Kakavas, Timothy E Hewett, Vasileios Korakakis

**Affiliations:** 1 Medicine and Research for High-Performance Sports, Centre Départemental de Formation en Activités Sportives (CDFAS), Eaubonne, FRA; 2 Research and Development, Société Française des Masseurs Kinésithérapeutes du Sport (SFMKS) Lab, Pierrefitte sur Seine, FRA; 3 Orthopedic Surgery, Clinic of Domont, Domont, FRA; 4 Sport Medicine, Orthosport Rehab Center, Domont, FRA; 5 Spine and Sports Laboratory, Fysiotek, Athens, GRC; 6 Orthopedic Surgery, Marshall University, Huttington, USA; 7 Physiotherapy, King's College, London, GBR

**Keywords:** bridging therapy, low load, elderly, blood flow restriction, total knee arthroplasty

## Abstract

Total knee arthroplasty (TKA) is one of the most performed operations in the world, especially in the elderly. Aging has a significant effect on joint cartilage, muscle strength, and muscle mass. Following a TKA, despite the significant reduction of symptoms and the improvement in mobility, muscle strength and muscle mass recovery remains a significant challenge. Restrictions that arise from the surgical procedure include joint loading, functional activities, and range of motion, along with limitations related to the age of the individual and their previous loading history, these are the significant restrictions, at least in the early stages of rehabilitation. Evidence indicates that blood flow restriction (BFR) training has significant potential to enhance recovery via implementation of low-load or low-intensity exercise. While respecting the indications and contraindications related to BFR application, the optimization of metabolic stress seems to offer a bridging therapy to heavy load while reducing pain and inflammation. Thus, the combination of BFR and low loads may improve muscular recovery (strength and mass), and aerobic training protocols appear to show significant enhancement of multiple cardiopulmonary parameters. Mounting evidence, direct and indirect, indicate that BFR training may have the potential to benefit the pre-operative and post-operative TKA rehabilitation phases and enhance functional recovery and physical abilities in the elderly.

## Introduction and background

Osteoarthritis is one of the leading causes of disability, with the knee being by far the most frequently affected joint [1]. Total knee arthroplasty (TKA) is a steadily increasing surgery in older populations and is considered as an effective management approach at the advanced stage of the condition [2]. Pain and muscle weakness and muscular atrophy are notable consequences of both osteoarthritis and surgery but for different reasons [3-7]. In addition, several other factors, such as sarcopenia or low physical activity, related to the elderly population may affect the outcome of TKA in terms of activities of daily living and quality of life [5,8,9]. Pre-habilitation, early post-operative physiotherapy, and progressive loading would appear to be essential to prevent these consequences, but exercise loading pain seems to be a potential limiting factor [10-13]. To optimize the relationship between loading and benefit in this population and promote and enhance recovery, effective treatments incorporating low loads and low exercise intensities must be implemented.

Contemporary evidence indicates that blood flow restriction training (BFR) has the potential to drive such improvements. By partial occlusion of the arteriovenous circulation and performance of low-load and low-intensity exercise, BFR training has been shown to significantly improve muscle strength and muscle mass [14-19]. In addition, BFR training has systemic effects (e.g., endocrine, cardiopulmonary, osteoblastic) that have been linked to functional improvements of the elderly, such as walking capacity [20,21], endurance [22,23], and reduction in risk of falls [22,24,25].

From a clinical perspective, BFR training has a strong potential in TKA rehabilitation and it is essential for its clinical use to consider an overview of indications and contraindications, as well as a guideline for its application in the elderly population following a TKA.

## Review

Indication

TKA significantly reduces pain and restores joint function of the knee, allowing patients to regain autonomy to perform activities of daily living [26]. However, significant muscle wasting, reduced muscle activation, and pain may be present after TKA and persist for several years [26,27]. The physiological requirements to drive and enhance muscle adaptations are to stress the muscle tissue and increase the demands of the neural drive at moderate to heavy loads during rehabilitation after TKA [28]. Rehabilitation includes pain-reducing interventions, as well as exercises to improve clinical musculoskeletal conditions or conditions managed operatively. Nevertheless, the effect of exercises may be attenuated in the presence of pain, where resistance training can be inhibited [29,30]. Evidence indicates that low-load resistance training with BFR can induce muscle hypertrophy and improve strength in healthy individuals and also indicates a moderate effect of low-load resistance training with BFR (LLRT-BFR) on enhancement of muscle strength in individuals suffering musculoskeletal weakness [31,32]. In addition, similar effects have been reported in patients with patellofemoral pain [33], in patients after Achilles tendon rupture [34], in patients with knee osteoarthritis [35], in the elderly [36], in patients after anterior cruciate ligament reconstruction [37], in patients after knee arthroscopy [21], in women with risk factors for knee osteoarthritis [38], and finally, has been reported to attenuate disuse atrophy during periods of immobilization [39]. Hence, BFR training would be a suitable tool to obtain these physiological benefits and adaptations in patients with knee osteoarthritis or TKA. The pain-modulating effect in conjunction with low stress applied to the tissues and joints during BFR training is a promising and effective option in TKA post-operative rehabilitation management [40].

Contraindications

BFR training is acknowledged to offer significant neuromuscular benefits and adaptations; however, it can possibly lead to side effects (e.g., numbness, nausea, headache, fainting, tingling) and there are potential significant adverse events (e.g., hypertension, venous thrombus, deterioration of ischemic heart disease, central retina vein occlusion, and rhabdomyolysis) if applied inappropriately. These occurrences are rare in clinical practice - with embolism being chief among them, but have been previously documented and a risk stratification recommendation has been published to guide safety in clinical practice [41]. There is no exhaustive list of absolute or relative BFR training contraindications, but possible BFR contraindications are considered self-evident conditions like cardiovascular diseases (e.g., coronary heart diseases, unstable hypertension, peripheral vascular disease, venous thromboembolism cardiopulmonary conditions, hemophilia), severe musculoskeletal injuries (e.g., recent muscle trauma or crush injuries, post-surgical excess swelling, skin graft, extremity infection), lifestyle conditions (e.g., pregnancy, smoking, obesity, uncontrolled diabetes mellitus), family medical history (e.g., clotting disorders, sickle cell anemia, atrial fibrillation or heart failure, cancer), and medications (e.g., known to increase blood clotting risk) [19,42].

No significant risk differences from BFR training have been reported for younger or older populations, while the only adverse events associated to individuals with knee osteoarthritis are related to exercise-induced knee pain [43]. Before and after TKA surgery, an older population with a modified or altered global physiological system should be adequately screened and associated risk factors should be identified before the commencement of rehabilitation. There is currently no evidence that indicates that BFR training is contraindicated after surgery given that all precautions with regard to the general application of BFR in rehabilitation are considered [44].

Safety in the application of BFR training has been extensively discussed in the literature, but to our knowledge, there are no established and validated criteria for its safe application in the operated elderly individuals [45]. Generic criteria and principles related to cardiovascular risk factors and comorbidity indicators to intense physical training have been proposed [45] in addition to stratified risk analysis [46]. Given that vascular restriction pressure exacerbates and fluctuates the hemodynamic and cardiovascular systems, an automated control of the pressure in real-time in patients at risk or post-operative patients has been suggested [46]. Contraindications for BFR application should be based on individualized evaluations and the interplay of factors, such as coagulation, hemodynamics, lifestyle, and medical history. In the postoperative context, the overall state of health of the patient and possible postoperative complications (defect or difficulty in healing, infection, inflammation, and increased pain) should be taken into account and the consensus of the medical team should be requested and obtained. We suggest that a standardized application of BFR training following TKA or other surgical procedures in elderly individuals is essential to minimize the risk of adverse events.

BFR training clinical application after TKA

The following modes of BFR training are based on narrative and systematic reviews that focused on populations with several knee joint-related conditions (after surgery or injury) or musculoskeletal rehabilitation (Table 1) [14,17-19,47]. BFR training mode of application (passive, BFR training with aerobic exercise, and BFR training with resistance exercise) varies in the literature and depends on clinical reasoning, safety, the specifics of the population (such as age, injury, surgery, concomitant issues, comorbidities, etc.), the characteristics of the condition (e.g., post-operative, phase of healing, stage of rehabilitation, etc.), and the aim of the application (e.g., strength or muscle mass improvement, pain reduction, improvement of aerobic capacity, etc.). The following modes of application have been adapted for patients following a TKA.

**Table 1:** Protocols for using BFR and effects. BFR: blood flow restriction; AT: aerobic training; LL: low load; MVIC: maximum voluntary isometric contraction; HL: heavy load; RM: resistance maximum; LOP: limb occlusion pressure; HRR: heart rate reserve; NMES: neuromuscular electrical stimulation

Variables	Passive BFR	BFR with aerobic exercise	BFR with low-load resistance training	BFR with high-load resistance training
Indication	Bedridden, immobilized, or weight-bearing patient	Mobile patient but not able to perform strengthening exercise	Patient able to perform strengthening exercise with low load	Patient able to perform exercises with high load (65-70% MVIC of the non-operative limb or pre-operative strength)
Exercises	Totally passive (no active contraction), possible addition of NMES	Walking or cycling (intensity <50% HRR)	Strengthening or functional exercise	Strengthening or functional exercise
Dosage	3-4 sets of 5 min occlusion/3 min without occlusion, 1-2 times/day	5-20 min of exercise (depending on the patient's abilities), 2-3 times/week	4 sets of 30-15-15-15 repetitions, 30 to 60s of rest, 20-40% MVIC, 2-3 times/week	4 sets of 30-15-15-15 repetitions, 30-60s of rest, 20-40% MVIC, 2-3 times/week, heavy-load exercises (70-80% RM)
LOP	70-100%	40-60%	40-60% (depending on the scope of training - pain or strength)	40-60%
Effects	Attenuate muscle atrophy, loss of muscle strength, and pain	Improve muscle strength and hypertrophy, attenuate pain	Improve muscle strength and hypertrophy, attenuate pain	Improve muscle strength and hypertrophy

Passive BFR training application

This mode of application is usually used at the beginning of rehabilitation when the patient is bedridden or is not yet able to perform muscular strengthening exercises with external load. The main effects of this mode are to prevent muscle atrophy and attenuate muscle strength decline during this period, but also to reduce post-operative pain and inflammation (the only BFR effects described in relation to inflammation are improvements in local mitochondrial and vascular functions) [17,18]. It has been argued that the passive mode of BFR application may promote a faster progression of the rehabilitation process [18].

With regard to the application, this mode does not involve active muscle contraction. The placement of the cuff is recommended at the most proximal part of the limb of interest [19]. There are reported applications in the literature that involve passive muscle contraction by using neuromuscular electrical stimulation in conjunction with BFR [14].

The suggested protocol for the passive BFR application aims toward maintenance of a limb occlusion pressure of 70-100% of the complete limb occlusion for five minutes and then three minutes of reperfusion (without occlusion). This protocol can be repeated three to four times per session (max time of occlusion 20 minutes per session) and it is advisable to be implemented once or twice a day [17,18].

BFR training application with aerobic exercise

This mode of BFR training is associated with an aerobic component that may be walking or cycling, depending on the knee range of motion available to the patient, surgeon’s instructions, and number of days post-surgery. This BFR training mode may be offered to patients who are not yet able to work with heavy load or high exercise intensities. The main objectives of this mode of application are mainly cardiorespiratory adaptations as well as gains in muscle strength if possible [17,18]. These effects would be due to a promotion of angiogenesis in response to intermittent localized hypoxia, which would thus play an important role in muscle adaptations. Careful selection of patients to be treated with this BFR application should be made, given that a significant percentage of elderly patients with knee osteoarthritis are likely to withdraw or discontinue BFR with aerobic exercise due to exercise-induced discomfort when using the cuff [48].

This BFR application protocol incorporates a limb occlusion pressure (LOP) of 40-80% of complete limb occlusion pressure during aerobic exercise for a period of 5-20 minutes for 2-3 times per week dependening on the patient's cardiorespiratory and musculoskeletal system capacity. Exercise intensity with BFR should not exceed 50% of the patient's heart rate reserve [17,18]. The specifics of the application are based on the patient's pain and tolerance, discomfort when using the cuff, and ability to perform the exercise.

BFR training with low- or high-load exercise

This mode of application can be offered to patients who possess the ability to perform resistance exercise with low or high external load and can be applied using strengthening machines or body weight functional exercise [18]. Low- or high-load BFR training aims at gaining muscle strength and muscle hypertrophy that are significantly more prominent than other BFR modes of application, especially compared to aerobics [49].

The standard BFR training with low- or high-load consists of four sets of exercise, where the first set of 30 repetitions is followed by three sets of 15 repetitions with 30-60 seconds of rest between sets with maintenance of the occlusive pressure to optimize metabolic stress. The load is suggested to be between 20% and 40% of the patient's maximum voluntary isometric contraction (assessed every two weeks with a hand-held isometric dynamometer at 90° of knee flexion). The exercise should be performed two to three per week, and should be paced by a metronome, (2 seconds concentric and 2 seconds eccentric phase, with limb occlusion pressure between 40% and 80%) [17,18,47,50,51].

If the patient is unable to perform the repetitions indicated by the training protocol, an adaptation of the rest time, a decrease in the load, a modification of the percentage of limb occlusion pressure, or the type of exercise should be performed. The loading protocol with BFR must be progressive in terms of load, percentage of limb occlusion pressure, and type of exercise according to the patient's abilities. It can thus be accompanied by strength training with high loads without BFR if the patient is able to perform an exercise without BFR with a load of 65-70% of the maximum voluntary isometric contraction without any adverse effects [18]. The muscular adaptations following this mode of BFR application would be comparable to a strengthening protocol based on heavy-load exercises between 70% and 80% of the maximum voluntary isometric contraction [18,50].

BFR systemic effects

Apart from the direct benefit of the use of BFR after TKA surgery as a means to improve muscle strength, several studies have placed their focus on systemic adaptation increases related to low-load (LL) training with this tool, which could help patients better recover or improve their quality of life throughout rehabilitation. In a systematic review from 2021, Miller et al. reported effects on three main components [52]. On the cardiopulmonary system, similar effects on blood pressure and heart rate were found in exercise with BFR as in traditional aerobic exercise. Maximal oxygen consumption (VO_2_ max) was also increased with BFR training and could even be observed with less training intensity and volume, which may not always be possible for patients immediately after TKA surgery due to swelling and inflammation-related pain. Vascular compliance has also been measured in studies reviewed by the authors, but more data is necessary to draw any conclusions on the effect of BFR on vascular adaptations. It is important to note that the protocols reported by the authors usually limit limb occlusion pressure to a maximum of 200 mmHg, as a higher pressure might not be in favor of better results.

Karabulut et al. reported an increase in bone alkaline phosphate (a serum bone marker related to osteoblastic activity) after six weeks of LL training with BFR in older adults, with results similar to a traditional high-intensity resistance training program [53]. This shows that osteoblastic activity can be increased even with lesser mechanical loading of the bones, which could help bone-prosthesis junction to consolidate in the first post-operative stages. This may also increase global bone mineral density, especially in populations often affected by osteoporosis but no evidence-based study is currently found [54,55].

The metabolic stress induced by training under BFR was also reported by Miller et al. as observed by an increase in blood lactate and growth hormone dosages, as well as a decrease in myostatin gene expression [52]. Although these changes are directly correlated with increased muscle mass and strength, it is important to note that systemic change will influence the whole body structure, including other limbs and in particular the contralateral leg. For example, Bowman et al. highlighted a crossover effect that benefited the non-involved leg [56]. In a context where most patients undergoing this type of surgery show signs of sarcopenia and loss of muscle strength, increased physiological capacity may prove beneficial as a way to prevent further loss of ability and activity. Reductions in the loss of strength on the contralateral limb may occur as well, especially in the early stage of rehabilitation, when the operative after-effects still limit patient activities. Although the use of LL resistance training with BFR mainly aims to increase specific muscle strength, the systemic changes induced may also be a secondary benefit to addition of this training method after TKA surgery [57].

## Conclusions

This article highlights that BFR training is a potentially effective adjunct to rehabilitation after TKA in the elderly. Based on the predicted rising amount of TKA surgeries, it is necessary to identify intervention strategies to reduce post-operative adverse effects. Through the shift from a mechanically to a more metabolically challenging exercise regime, BFR training appears to be a promising pre-habilitation and post-operative rehabilitation strategy. BFR training appears to improve strength and function during implementation of low aerobic exercise intensities and low load in strengthening exercise. Nevertheless, it is important to consider the indications, contraindications, and eligibility criteria of the patients in order to optimize the target parameters (muscular and aerobic) and more specifically the training mode, frequency, time under tension/load, occlusion pressure, frequency of exercise, and other patient-related or condition-related factors. Clinicians should implement BFR applications in elderly rehabilitation follow TKA with caution and following the evidence base, the safety considerations, their expertise, and their clinical reasoning. Further studies are still needed to confirm BFR efficiency in elderly people and observe its effects on a better return to activity and an increase in their quality of life.
